# Effects of domestication and captive breeding on reaction to moving objects: implications for avoidance behaviours of masu salmon *Oncorhynchus masou*

**DOI:** 10.1098/rsos.230045

**Published:** 2023-04-26

**Authors:** Koh Hasegawa, Masanori Nakae, Kouta Miyamoto

**Affiliations:** ^1^ Salmon Research Department, Fisheries Resources Institute, Japan Fisheries Research and Education Agency, Nakanoshima, Toyohira, Sapporo 062-0922, Japan; ^2^ Department of Zoology, National Museum of Nature and Science, 4-1-1 Amakubo, Tsukuba, Ibaraki 305-0005, Japan; ^3^ Nikko Field Station, Fisheries Technology Institute, Japan Fisheries Research and Education Agency, Nikko, Tochigi 321-1661, Japan

**Keywords:** hatchery programme, lateral line, learning, salmonid, wild

## Abstract

Domestication and captive breeding can compromise the obstacle- and predator-avoidance capabilities of animals in the wild. Whereas previous studies only examined these effects in combination, here we examine them individually by comparing the abilities of wild, F1 (offspring of wild parents) and captive-bred (approx. F15) masu salmon *Oncorhynchus masou* to avoid a falling object under experimental conditions. Rates of avoidance failure were low (wild, 12.5%; F1, 10.7%; captive-bred, 8%) under light conditions, but increased under dark conditions (wild, 11.1%; F1, 32.1%; captive-bred, 60.0%). We attribute the elevated avoidance failure rate among F1 fish to the lack of learning opportunities in hatchery environments (i.e. domestication), and the further elevation of avoidance failure rate among captive-bred fish to the degradation of sensory organ function (i.e. captive breeding). These results imply reduced survival rates for F1 and captive-bred fish in the wild and are consistent with the low stocking efficiencies reported for captive-bred masu salmon.

## 1. Introduction

Avoidance behaviours for threats such as predators and approaching obstacles with the potential to cause physical damage are a very common adaptation among animal species [1,2]. Patterns of avoidance behaviours vary not only among but also within species by the type of threat, habitat condition (e.g. visibility) and personality [3,4]. Properly functioning sensory organs are required to recognize approaching objects that necessitate avoidance [5,6]. Learning opportunities can also lead to the acquisition of avoidance behaviours [7].

Captive-bred animals are usually domesticated in artificial environments where, in contrast with natural environments, there are very few threats to their survival [8]. Adaptation to such environments can involve the degradation of sensory organs in captive-bred animals [9,10] and could weaken avoidance behaviour by delaying the perception of approaching objects. Also, domestication in artificial environments reduces opportunities to learn how to identify threats that animals must avoid [11]. Because domestication and captive breeding generally affect animals simultaneously, previous studies have not separately examined the effects of these two factors.

Salmonid fishes are domesticated in rearing facilities (fish hatcheries) for fisheries enhancement and aquaculture across the world [12]. Captive breeding is conducted for stocking and aquaculture, while sexually mature fish are caught in natural populations to produce fry in some cases (so-called F1 fish that are also domesticated in a fish hatchery until stocking) [13]. In natural environments, salmonids avoid strong currents and obstacles carried by debris floods by escaping into rock shadows and nearby tributaries [14,15] and avoid both terrestrial and aquatic predators by hiding in shadows and swimming away [16]. In addition to being visually oriented [17], salmonids have lateral line systems to detect hydraulic pressure waves created by approaching objects [18].

Captive breeding is known to alter the behaviour of salmonid fishes [19], and both captive-bred and F1 fish are usually domesticated in fish hatcheries where there is a lack of opportunity to learn avoidance behaviour [20]. This means that a comparison of the two fish types could allow an examination of the effects of captive breeding separately from domestication. In our experiments, we tested the hypothesis that any alterations of avoidance behaviour are more pronounced in captive-bred fish than in F1 fish because captive-bred fish are affected by both factors. We conducted our experiments under light conditions, in which fish could rely on both vision and lateral lines, and under dark conditions, in which fish could only rely on lateral lines.

## 2. Methods and materials

### 2.1. Model species

Masu salmon *Oncorhynchus masou* fry of a strain originating from the Shiribetsu River in Hokkaido, Japan was used for the experiment. Captive-bred fish were reared in the fish hatchery at the Nikko Field Station, Japan Fisheries Research and Education Agency (FRA) for approximately 15 generations. These fish were reared for three months in an outdoor columnar tank (approx. 300 fish; diameter 105 cm, depth 48 cm) equipped with a cover and anti-animal netting to prevent intrusion by avian and mammalian predators. F1 fish were obtained from anadromous males and females captured during spawning migrations in the Mena River, a tributary of the Shiribetsu River. These fish were reared in an indoor artificial pond (approx. 100 000 fish; pond length 19 m, width 1.7 m, depth 0.4 m) at the Rankoshi Fish Hatchery, the Shiribetsu Field Station, FRA, for six months. Although rearing conditions at the two fish hatcheries were not identical because of their different locations (e.g. differences in temperature and day length), rearing facilities and procedures (e.g. differences in fish density and feeding regimes), both fish hatcheries reared fish under natural sunlight and without exposure to predators and any other obstacles. Wild fish were caught in another small wadable tributary, the Sousuke stream (located at 42°52′52.2″ N, 140°45′3.9″ E) by electrofishing (Model 12B, Smith-Root Inc., Vancouver, WA, USA) in late June 2020. In the Sousuke stream, other sympatric salmonids (e.g. large masu salmon, rainbow trout *Oncorhynchus mykiss*, brown trout *Salmo trutta*) and avian species such as common kingfisher *Alcedo atthis* are potential predators of masu salmon. The river is surrounded by broadleaf trees, which are a source of fallen trunks and branches and other obstacles that masu salmon would need to avoid. The age in months of the wild fish was unclear. However, masu salmon in Hokkaido usually start to emerge in streams from March to April (e.g. [21,22,23]). Because masu salmon is an important species for recreational inland fisheries and commercial coastal fisheries, captive-bred and F1 fish are stocked into many rivers in northern Japan, but the contribution of these fish to resource enhancement is uncertain [24,25].

### 2.2. Behavioural observations in light and dark conditions

Behavioural observations were conducted in an aquarium (length 45 cm, width 27 cm, height 30 cm) in a laboratory at the Salmon Research Department, Fisheries Resources Institute, FRA. Opaque sheets were used to block any incoming natural light, and fluorescent lights were used for illumination. A thin plastic plate was used to separate the aquarium into two sections: a 17 cm wide back section and a 10 cm wide front section. Well water was gently poured into the back section to minimize any water flow in the front section and maintain the water depth at 21 cm. The water temperature was maintained at a suitable level (10.5–13.0°C) for behavioural observation of salmonids. Thus, we assumed the datal difference of the experiment for each origin of fish was negligible for interpreting the results (table 1).

**Table 1.** Number (*n*) and fork length (FL; mm) (top: mean ± s.d.; bottom: range) of experimental fish from each origin tested under light and dark conditions. ‘period’ indicates the dates when behavioural observations were conducted.

origin	light		dark		period
	*n*	FL	*n*	FL	
captive-bred	25	48.1 ± 3.0 42–53	30	48.3 ± 2.5 43–54	20–24 February 2020
F1	28	53.2 ± 4.8 44–61	28	53.1 ± 3.9 46–61	2–5 June 2021
wild	24	54.4 ± 3.9 46–60	27	54.0 ± 3.9 48–61	23–26 June 2020

One experimental fish at a time was selected haphazardly from either of two aquaria used to hold experimental fish (length 60 cm, width 30 cm, height 36 cm), caught by using a small dip net and transferred into the front section of the observation aquarium. The two aquaria used for holding fish were filled with the same well water as the aquarium used for behavioural observation. Fewer than 30 fish were kept in each of the two aquaria beginning a few days before the experiments and they were fed a few pinches of pellets daily. The experimental fish were visually healthy (i.e. had no visible injuries from aggressive interactions with conspecifics or collisions with aquarium walls, and did not appear to be emaciated from lack of food) during the experiments.

**Figure 1.**
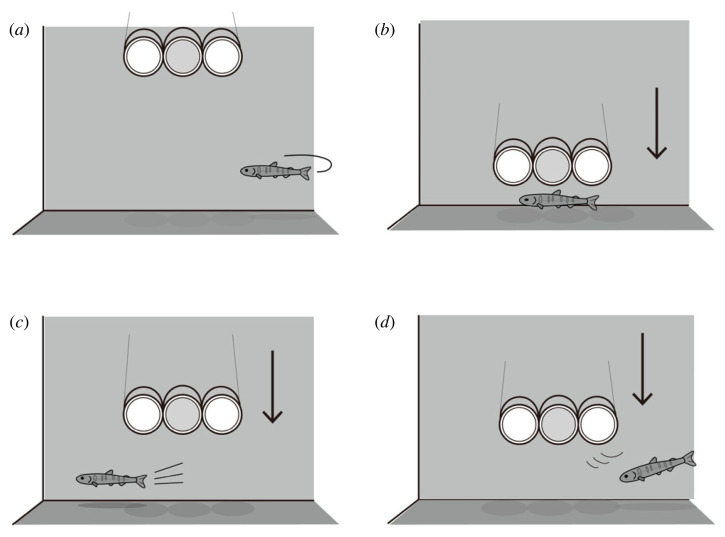
Diagrams of fish behaviours observed in this study. The panels show (*a*) the experimental fish prior to release of the falling object, (*b*) avoidance failure, (*c*) successful avoidance through forward swimming and (*d*) successful avoidance through freezing.

Avoidance behaviour was triggered by using a falling object. The object (three interlocked cylinders, cylinder diameter 5.5 cm, height 9 cm and weight 230 g) was suspended on a nylon line at the centre of the aquarium such that the top of the object was positioned immediately below the water surface (figure 1*a*). One end of the nylon line was fastened to a clasp to immobilize the object. When the experimental fish was at a depth greater than 14 cm (i.e. two-thirds of the depth of the aquarium) and its snout was located directly beneath the object, the nylon line was released to allow the object to fall silently. The object was released a single time for each experimental fish.

Observations were conducted both under light conditions (approx. 300 lx) and dark conditions (0 lx). The experiments under dark conditions were used to determine whether any differences in avoidance behaviour among captive-bred, F1 and wild fish were caused by differences in their lateral line systems. The sequencing of light and dark conditions was random. Acclimation time to the aquarium was 20 min. We confirmed that the experimental fish did not sleep (i.e. become motionless) during this time. Each experiment was recorded by an infrared video camera (CMS-SC01GY, SANWA SUPPLY Inc., Okayama, Japan). Under dark conditions, fish locomotion was observed by using a night-vision scope (ZIYOUHU NV-2000C, Zhejiang ZIYOUHU Optoelectronic Technology Co., Ltd, Sichuan, China) and 940 nm infrared light (EnergyPower SA3-IR, World Energy Trading Co., Ltd, Kowloon, Hong Kong). After each observation, the number of lateral line organs was counted. The lateral line organs on both the head and body were counted on fish stained with DiAsp and anaesthetized (and euthanized) with a *ca* 0.1% solution of FA 100 (4-Allyl-2-methoxyphenol: eugenol) (see [26] for detailed procedures). The number of replicates, the body size of each experimental fish and the experimental periods are summarized in table 1. Water temperature and illuminance were recorded by a floating data logger placed in the back section of the aquarium (HOBO MX2201, Onset Computer Co., Bourne, MA, USA).

### 2.3. Data analysis

Differences in the number of lateral line organs (in total, on the head and on the body) on captive-bred, F1 and wild fish were tested by using two-way ANOVA followed by Scheffe's tests. The ANOVAs considered origin, light/dark condition and an interaction term as explanatory variables. The numbers of lateral line organs were log_10_-transformed.

Observed behaviours were classified into three patterns: avoidance failure, defined as the object directly impacting the fish (figure 1*b*; electronic supplementary material, S1); avoidance by forward swimming (figure 1*c*; electronic supplementary material, S2) and avoidance by freezing, defined as the object missing the fish because the fish stopped swimming before the object reached it [27] (figure 1*d*; electronic supplementary material, S3). The latter two patterns were regarded as successful avoidance behaviour.

Failure or success of avoidance for each fish origin (captive-bred, F1 and wild) under light and dark conditions were compared by using a generalized linear mixed model (GLMM) with observation as a random effect, as follows:

$$\begin{array}{rl} \mathrm{Failure}/\mathrm{success}=\; & \mathrm{origin}+\mathrm{light}/\mathrm{dark}+\mathrm{number}\;\mathrm{of}\;\mathrm{lateral}\;\mathrm{line}\;\mathrm{organs}\;(\mathrm{no}.\;\mathrm{of}\;\mathrm{organs})\\ & +\mathrm{origin}\times \mathrm{light}/\mathrm{dark}+\mathrm{origin}\times \mathrm{light}/\mathrm{dark}\times \mathrm{no}.\;\mathrm{of}\;\mathrm{organs}. \end{array}$$

For successful avoidance behaviours, the behavioural pattern used (i.e. forward swimming or freezing) was compared in the same manner:

$$\begin{array}{rl} \mathrm{Behavioural}\;\mathrm{pattern}=\; & \mathrm{origin}+\mathrm{light}/\mathrm{dark}+\mathrm{no}.\;\mathrm{of}\;\mathrm{organs}\;\\ & \;+\mathrm{origin}\times \mathrm{light}/\mathrm{dark}+\mathrm{origin}\times \mathrm{light}/\mathrm{dark}\times \mathrm{no}.\;\mathrm{of}\;\mathrm{organs}. \end{array}$$

A binomial distribution with logit link function was assumed for these GLMMs. If the highest order interaction term was insignificant, GLMM analysis was repeated without that interaction term.

Statistical tests were performed in SPSS version 24 (IBM Corp., Armonk, NY, USA). The alpha was set to 0.05.

## 3. Results

**Figure 2.**
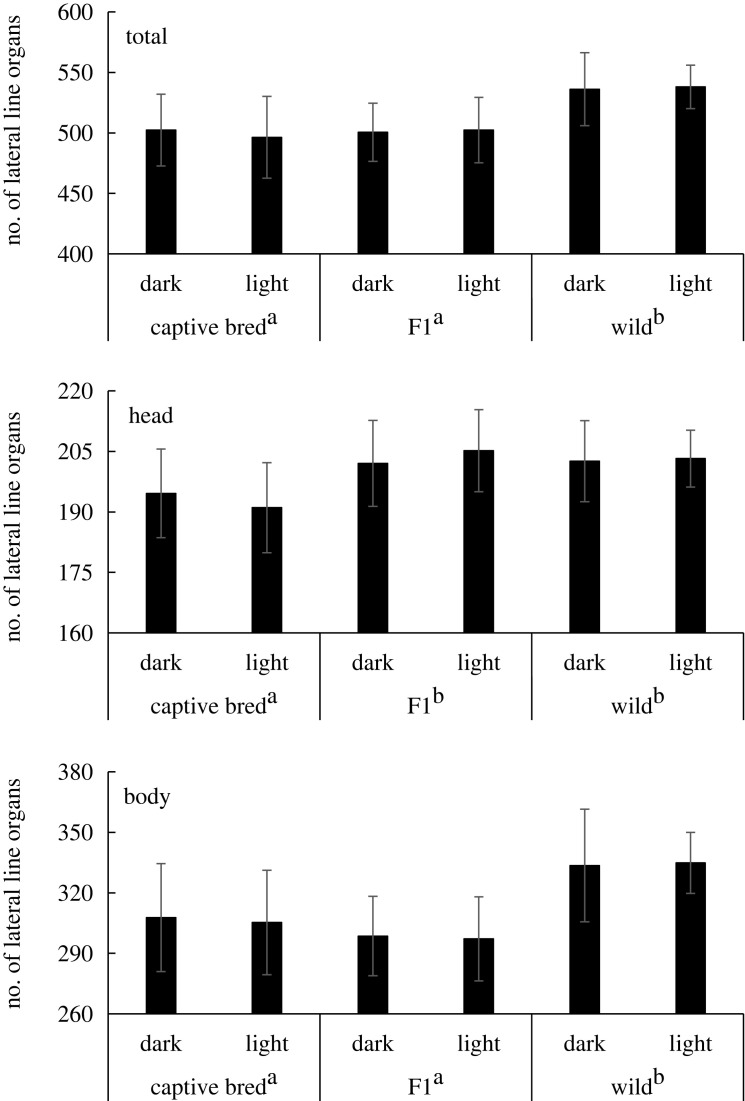
Mean number of lateral line organs (in total, on the head and on the body) of captive-bred, F1 and wild fish used in the experiments. Origins that do not share a superscript letter are significantly different (two-way ANOVA followed by Scheffe's tests). Error bars show s.d.

The number of lateral line organs on the heads of captive-bred fish was smaller than on those of F1 and wild fish, and the number on the bodies of captive-bred and F1 fish was smaller than that on wild fish. The total number of lateral line organs of captive-bred and F1 fish was smaller than that of wild fish (figure 2 and table 2). However, the total number of lateral line organs had no significant effect on avoidance failure/success or on patterns of successful avoidance behaviour (tables 3 and 4).

**Table 2.** Summary of two-way ANOVAs for testing the differences in numbers of lateral line organs (in total, on the head and body) of experimental fish among origins and between light and dark conditions in the experimental aquarium.

	d.f.	*F*	*p*
total			
origin	2	28.291	<0.001
light/dark	1	0.018	0.893
origin × light/dark	2	0.352	0.704
head			
origin	2	18.664	<0.001
light/dark	1	0.004	0.948
origin × light/dark	2	1.526	0.221
body			
origin	2	30.405	<0.001
light/dark	1	0.036	0.849
origin × light/dark	2	0.078	0.925

**Table 3.** Summary of GLMM analysis for avoidance failure/success. ‘d.f.1’ and ‘d.f.2’ indicate the effect degrees of freedom and the error degrees of freedom, respectively.

	d.f.1	d.f.2	*F*	*p*
origin	2	155	0.889	0.413
light/dark	1	155	8.401	0.004
no. of organs^a^	1	155	0.340	0.561
origin × light/dark	2	155	3.085	0.049
origin × light/dark × no. of organs	5	150	1.029	0.403

^a^Number of lateral line organs.

**Table 4.** Summary of GLMM analysis for patterns of successful avoidance behaviour. ‘d.f.1’ and ‘d.f.2’ indicate the effect degrees of freedom and the error degrees of freedom, respectively.

	d.f.1	d.f.2	*F*	*p*
origin	2	119	5.098	0.008
light/dark	1	119	39.92	<0.001
no. of organs^a^	1	119	1.715	0.193
origin × light/dark	2	117	1.080	0.343
origin × light/dark × no. of organs	5	112	0.358	0.876

^a^Number of lateral line organs.

**Figure 3.**
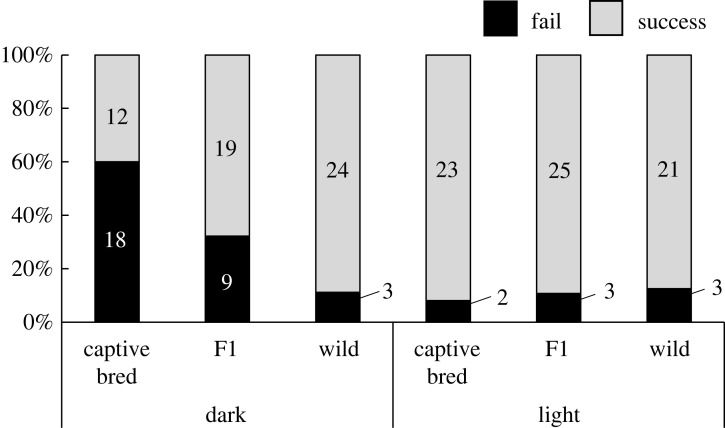
Percentages of failed (black) and successful (grey) avoidance behaviours for captive-bred, F1 and wild fish under light and dark conditions. Numbers in bars are the number of failed or successful avoidance behaviours observed.

A significant interaction between origin and light/dark was detected for avoidance failure/success (table 3). Experimental fish of all origins seldom failed to avoid the falling object under light conditions (wild, 12.5%; F1, 10.7%; captive-bred, 8%), but were more prone to avoidance failure under dark conditions, with failure rates being lowest for wild fish (11.1%), intermediate for F1 fish (32.1%) and highest for captive-bred fish (60.0%) (figure 3).

**Figure 4.**
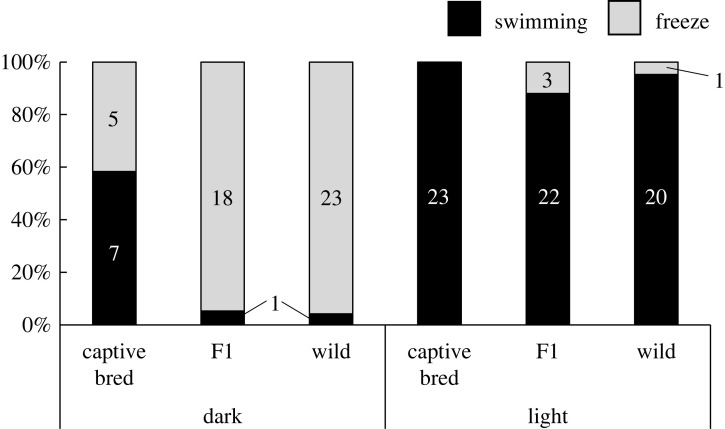
Percentages of successful avoidance behaviours that occurred from forward swimming (black) and freezing (grey) among captive-bred, F1 and wild fish under light and dark conditions. Numbers in bars are the number of forward swimming or freezing behaviours observed.

Additionally, origin and light/dark condition were both found to have significant effects on patterns of successful avoidance behaviour (table 4). Captive-bred fish were less likely to show freezing behaviour than F1 and wild fish (figure 4). For all fish, forward swimming dominated under light conditions, whereas freezing was predominant under dark conditions (figure 4).

The statistical comparisons by GLMMs described above were not substantially different when the total number of lateral line organs was replaced by the numbers on the head or body.

## 4. Discussion

Our results show that the rate of avoidance failure was similarly low (maximum 12%) among fish of different origins under light conditions, but increased in the order of wild, F1 and captive-bred fish under dark conditions. Therefore, our initial hypothesis that alterations of avoidance behaviour were more pronounced in captive-bred fish than in F1 fish was only supported under dark conditions.

The rate of avoidance failure among F1 fish, which are not affected by captive breeding but domesticated, was intermediate between that of wild and captive-bred fish under dark conditions. Given this, we speculate that a lack of opportunities to learn avoidance behaviour can increase avoidance failure to some extent. The higher degree of avoidance failure among captive-bred fish under dark conditions may reflect degradation of the lateral line system through captive breeding, in addition to the lack of learning opportunities by domestication. In contrast with the results reported by Nakae *et al*. [28], our results do not show a clear reduction in the number of lateral line organs on captive-bred fish as compared with F1 and wild fish. However, our results do show that captive-bred fish reacted more slowly to falling objects than did F1 and wild fish. Thus, we speculate that the transmission of information from the lateral lines to the brain might be impaired in captive-bred fish. Although avoidance rates differed among fish groups under dark conditions, most fish regardless of origin successfully avoided the falling objects under light conditions. This suggests that domestication and captive breeding does not influence the visual acuity of masu salmon, and vision proved more effective for detecting approaching obstacles than lateral line organs under light conditions. Note that differences in the rearing conditions and ages of fish among the three origins could have influenced the behaviour of the experimental fish in addition to the factors we discussed in this paragraph.

The differences of avoidance behaviour that we identified among captive-bred, F1 and wild fish could cause differences in other behaviours. For example, some reports have found that captive-bred salmon smolts tend to undertake seaward migration during the day (e.g. Atlantic salmon *Salmo salar* [29]; masu salmon [30]), in contrast with F1 or wild salmon, which tend to migrate at night (e.g. Atlantic salmon [29]; chum salmon *Oncorhynchus keta* [31]). Although salmon generally migrate toward the sea at night to avoid encounters with diurnal predators [32], captive-bred and F1 fish might be forced into daytime migration due to their inferior avoidance success under dark conditions.

To avoid the falling object, the experimental fish generally swam forward under light conditions and froze under dark conditions. For example, three-spined stickleback *Gasterosteus aculeatus* becomes less active in more turbid habitats (i.e. in conditions that are less amenable to vision) to decrease the risk of encountering threats, although the authors did not describe how the behavioural patterns in these habitats compare with those in less turbid habitats [33]. In a similar way, the avoidance success rate associated with movement away from the threat (i.e. forward swimming in this experiment) might be higher than that associated with staying in place (i.e. freezing) only if the fish can visually interpret its surroundings. Under dark conditions, moving could entail a heightened risk of encountering another threat for visually oriented species, making freezing a viable alternative behaviour for threat avoidance. However, the reason that freezing behaviour was rarer among captive-bred fish than among F1 and wild fish is unclear. Although the sample size for successful avoidance was not large (*n* = 12; figure 4), we cannot deny the possibility that captive breeding alters patterns of avoidance behaviour, as has been reported for other behaviours (e.g. competitive and migratory behaviours [34]).

In this study, we were unable to detect any effects of the number of lateral line organs on avoidance failure rates or on patterns of successful avoidance behaviour. However, this should not be taken as definitive evidence that the number of lateral line organs is unrelated to avoidance behaviour. Although we used objects of uniform weight in our experiments, some uncertainty in our results could reflect variations in falling speed depending on the depth at which the experimental fish encountered the falling object. In addition, it is possible that the number of lateral line organs can affect avoidance behaviours at falling speeds outside the range examined in our study.

We could not identify what the experimental fish interpreted the falling object to be in this study. Although most previous studies have treated moving objects as simulated predators regardless of the taxonomic group of the animals [2], fish in natural environments need to avoid not only predators but also obstacles such as sediment flow. Further studies are required to test what fish recognize approaching objects as, and whether domestication affects this recognition ability.

Captive-bred and F1 individuals are sometimes released into natural environments to increase the stock size of biological resources or to conserve endangered populations [35–37]. However, our results suggest that the release of captive-bred individuals may be less efficient than the release of F1 individuals, because the survival rates of captive-bred individuals is compromised by their poor avoidance behaviour. Additionally, it is possible that other important behavioural traits are altered by these changes in avoidance behaviour. In fact, survival rates of captive-bred masu salmon are lower than that of F1 fish [38], and it is suggested that predator avoidance under low-light conditions is one factor that might influence survival rate [39]. Therefore, management schemes that rely on the stocking of captive-bred individuals should take into account the potential for reduced survival rates in natural environments caused by declines in functions of sensory organs and related changes to behaviour.

## Data Availability

The dataset has been submitted to Dryad (doi:10.5061/dryad.x95x69pnz) [40]. The videos are available in the electronic supplementary material [41].
